# Contamination of equipment and surfaces in the operating room anesthesia workspace: a cross-sectional study

**DOI:** 10.1590/1516-3180.2023.0177.R1.291123

**Published:** 2024-02-23

**Authors:** Carlos Eduardo Macedo, Adriano Menis Ferreira, Larissa da Silva Barcelos, André Luiz Silva Alvim, Liliane Moretti Carneiro, Sandro Rogério Martins, Denise de Andrade, Marcelo Alessandro Rigotti, Ruberval Peres Gasques, Vanderlei Amaro da Silva, Layze Braz de Oliveira, Herica Emilia Félix de Carvalho, Alvaro Francisco Lopes de Sousa

**Affiliations:** I MD. Physician, Postgraduate Program in Nursing, Universidade Federal de Mato Grosso do Sul (UFMS), Campo Grande (MS), Brazil.; II PhD. Nurse, Full Professor, Postgraduate Program in Nursing, Universidade Federal de Mato Grosso do Sul (UFMS), Três Lagoas (MS), Brazil.; III PhD. Associate Professor, Postgraduate Program in Nursing, Universidade Federal de Mato Grosso do Sul (UFMS), Três Lagoas (MS), Brazil.; IV PhD. Associate Professor, Graduate Program in Nursing, Universidade Federal de Juiz de Fora (UFJF), Juiz de Fora (MG), Brazil.; V MSc, Nurse, Doctoral Student, Postgraduate Program in Nursing, Universidade Federal de Mato Grosso do Sul (UFMS), Campo Grande (MS), Brazil.; VI MD. Physician, Hospital Regional de Presidente Prudente (SP), Brazil.; VII PhD. Nurse, Full Professor, Ribeirão Preto College of Nursing, Universidade de São Paulo (USP), Ribeirão Preto, São Paulo (SP), Brazil.; VIII PhD. Nurse, Associate Professor, Postgraduate Program in Nursing, Universidade Federal de Mato Grosso do Sul (UFMS), Três Lagoas (MS), Brazil.; IX Nurse. Master Student, Postgraduate Program in Nursing, Universidade Federal de Mato Grosso do Sul (UFMS), Três Lagoas (MS), Brazil.; X BS. Biomedic, Postgraduate Program in Nursing, Universidade Federal de Mato Grosso do Sul (UFMS), Campo Grande (MS), Brazil.; XI PhD, Nurse, Ribeirão Preto College of Nursing, Universidade de São Paulo (USP), Ribeirão Preto, São Paulo (SP), Brazil.; XII PhD. Nurse, Ribeirão Preto College of Nursing, Universidade de São Paulo (USP), Ribeirão Preto, São Paulo (SP), Brazil.; XIII PhD. Nurse, Institute of Teaching and Research, Hospital Sírio-Libânes, SP, Brazil.

**Keywords:** Infection control, Anesthesia, general, Equipment contamination, Patient safety, Colonization, Microorganisms, Mechanical ventilation, Cleaning and disinfection, Breathing circuit

## Abstract

**BACKGROUND:**

Contamination of the breathing circuit and medication preparation surface of an anesthesia machine can increase the risk of cross-infection.

**OBJECTIVE:**

To evaluate the contamination of the anesthetic medication preparation surface, respiratory circuits, and devices used in general anesthesia with assisted mechanical ventilation.

**DESIGN AND SETTING:**

Cross-sectional, quantitative study conducted at the surgical center of a philanthropic hospital, of medium complexity located in the municipality of Três Lagoas, in the eastern region of the State of Mato Grosso do Sul.

**METHODS:**

Eighty-two microbiological samples were collected from the breathing circuits. After repeating the samples in different culture media, 328 analyses were performed.

**RESULTS:**

A higher occurrence of *E. coli, Enterobacter spp., Pseudomonas spp., Staphylococcus aureus, and Streptococcus pneumoniae* (P < 0.001) were observed. Variations were observed depending on the culture medium and sample collection site.

**CONCLUSION:**

The study findings underscore the inadequate disinfection of the inspiratory and expiratory branches, highlighting the importance of stringent cleaning and disinfection of high-touch surfaces.

## INTRODUCTION

The transmission of microorganisms in healthcare settings, including perioperative areas, has become a potential concern in recent studies. The occupancy of spaces by previously colonized or infected patients increases the risk of acquiring multidrug-resistant microorganisms, emphasizing the critical importance of robust cleaning and disinfection procedures.^1-2^ The persistence of contamination on environmental surfaces has been linked to insufficient cleaning practices in operating rooms and anesthesia workspaces. This is of particular concern as eliminating multi-drug-resistant microorganisms from these surfaces is a key strategy to mitigate the prevalence of healthcare-associated infections (HAIs) that significantly contribute to morbidity, mortality, and extended hospital stays.^2-5^

The existing^3-6^ cleaning practices in operating rooms and anesthesia work areas are inadequate; therefore, environmental surfaces remain contaminated. Removing multidrug-resistant microorganisms from operating room surfaces is essential to minimizing the risk of HAIs. Furthermore, HAIs contribute to major public health problems by increasing morbidity and mortality rates and prolonging the hospitalization time of patients.^1-5^

In particular, surgical patients with open wounds are at a higher risk;^6^ therefore, the potential for cross-transmission in the intraoperative environment poses a threat to patient safety.^7^ Contamination in the anesthesia work area, including the anesthesia cart, faucets, laryngeal masks, laryngoscope blades, touchscreens, keyboards, and the hands of professionals, can result in the transmission of infections that promote health risks, highlighting pneumonia associated with mechanical ventilation (PAVM).^1-4,8^

Ventilator-associated pneumonia (VAP) is characterized as an infectious disease with an imprecise diagnosis and multiple causes, which allows divergent recommendations related to preventive measures, diagnosis, and treatment.^9-11^ Because 24 h of intubation in invasive mechanical ventilation (IMV) favor the colonization of microorganisms in the lower airways, orotracheal intubation performed during surgeries that require general anesthesia may also be a risk factor for PAVM.^10-11^

Despite practicing high-level cleaning and disinfection protocols, breathing circuits and anesthesia cart surfaces are contaminated by microorganisms such as gram-negative bacteria.^10-15^ Patients undergoing general anesthesia with IMV have variable health conditions, which increases the risk of cross-infection in the intraoperative period due to contamination of the respiratory circuit and the surface for medication preparation.^16^

However, despite advances in addressing HAIs, substantial gaps in understanding the dynamics of contamination persist within the confines of operating rooms as well as the intricate landscape of anesthesia-related equipment.^1-3^ Of these gaps, the precise level of contamination in operating rooms, more specifically, surfaces designated for the preparation of anesthetic drugs, is of particular concern. Similarly, anesthesia-related devices, which are crucial components of patient care, hold utmost importance in this context, necessitating a deeper exploration of their potential roles in transmitting HAIs in this environment ^2-5,8^

Comprehensive studies investigating operating room environments, especially those involving the intricate interaction of instrumental surfaces in the preparation of anesthetic drugs, are lacking. Therefore, constructing a comprehensive picture of the persistent contamination levels in these critical areas remains challenging. Although commendable efforts have been made to develop guidelines for reducing HAIs, their implementation and adoption in the surgical environment are inconsistent. This could lead to a variation in infection prevention practices, which increases the possibility of potential oversights that could compromise patient safety.^2,4-5,17-19^

Furthermore, the shortage of routine compliance audits exacerbates this issue. The potential for misguided practices increases owing to the lack of regular and rigorous evaluation of adherence to infection prevention measures.

## OBJECTIVE

The objective of this study was to evaluate the contamination of anesthetic medication preparation surfaces, respiratory circuits, and devices used in general anesthesia with assisted mechanical ventilation.

## METHODS

This was a cross-sectional, quantitative study conducted at the surgical center of a philanthropic hospital of medium complexity, located in the municipality of Três Lagoas, in the eastern region of the State of Mato Grosso do Sul. This research was derived from a master’s thesis submitted to the graduate program in Nursing at the Federal University of Mato Grosso do Sul.^14^

The institution has 188 active beds, 60% of which are allocated to the Unified Health System (in Portuguese, Sistema Único de Saúde, SUS). This facility has been used for teaching, research, and extension purposes for the students at the Universidade Federal do Mato Grosso do Sul (UFMS) for more than 20 years. The surgical center has four operating rooms for elective, non-stop urgent, and emergency surgeries.

The study center did not have a protocol for changing the breathing circuit of the anesthesia machine, and some equipment had heat and humidity exchange filters (HMEF), whereas others did not. Devices that do not use an HMEF have breathing circuits that were changed after each surgery involving IMV. For devices with a filter, only the filter was changed after each procedure.

Additionally, the institution’s protocols and practices for processing the respiratory circuit of the anesthesia machine are not performed using a single method. In general, high-level disinfection was performed using autoclaves at 121°C and/or 134°C or automatic thermo-disinfector washers.

The exchange of components between the common gas outlet and patient (corrugated tubes, inspiratory branch, expiratory branch of the circuit, Y-piece, and connectors) is only performed when a bacterial filter is not used in general inhalatory anesthesia. Notably, this filter was placed between the anesthesia equipment and the patient’s airways to prevent postoperative pneumonia.

The requirement for ethics committee approval was waived because this study did not involve human participants and included only surfaces that make up the anesthetist’s work area, namely the anesthesia machine and the surface for drug preparation. Prior to the coronavirus disease 2019 (COVID-19) pandemic, approximately 500 surgical procedures every month were performed in this hospital, of which approximately 150 used IMV. However, data were collected during the severe acute respiratory syndrome coronavirus 2 pandemic, which directly affected the final samples obtained.

Respiratory circuits and surfaces in the anesthetic medication preparation area used in surgeries with indications for general anesthesia with IMV were considered eligible for collection of microbiological material. These surfaces were selected for analysis because they were frequently touched by the hands.

Breathing circuits and drug preparation surfaces used in surgeries for previously diagnosed lung disease and/or orotracheal intubation performed outside the surgical center were excluded.

Data were collected between August and September 2020 by the researchers themselves. Using non-probabilistic sampling for convenience, 82 samples were included for microbiological evaluation from four different locations, and a total of 328 analyses were obtained after repetition in different culture media.

In the first stage, samples were collected from the distal portion of the inspiratory branch before anesthesia; the distal portion of the expiratory branch after anesthesia; the breathing circuit canister at the end of anesthesia; and the surface of the anesthetic medication area before preparation, following a procedure recommended in the literature.^15^

The samples were obtained by performing circular friction with a sterile cotton swab, soaked in sterile saline solution, across the inner surface of the tracheas as far as the swab shaft reached. Circular movements were also used within the canister to apply friction from the sterile cotton swab to the internal walls of this device.^15^ Different swabs were used to collect samples from the inspiratory branch, the expiratory branch, the canister, and the surface of the anesthetic medication area.

After rubbing each component of the respiratory circuit of the anesthesia machine, the pre-molded lid of the transport tube, which made up the swab, was removed, and the cotton swab was submerged into Stuart’s transport medium. The transport tube was then identified based on the date, name of the surface collected, and the number that represented the collection. After the surgical procedure, three granules of soda lime were collected for each analyzed respiratory circuit, and stored in a sterile 60 mL plastic bag with a stripe and sealed. The plastic bags received an identification label with the date and collection representation number.^15^

All samples were packed in a box for transporting biological samples. The box was washable, resistant to disinfection and bearing the identification of “infectant” or “biological risk.” Subsequently, the samples were transported to the Laboratory of Microbiology and Molecular Genetics, located at the Universidade Federal do Mato Grosso do Sul, Campus of Três Lagoas, for processing.

In the first stage, Stuart medium was used as the transport medium to transport the samples to the microbiology laboratory. The Stuart medium is a semi-solid medium that contains thioglycolate, glycerol phosphate, and sodium chloride. Although it does not have a nutrient medium, the viability of most pathogens can be preserved. Because of the non-uniformity of the studied components, the minimum and/or maximum surface for collection was not defined, and friction was carried out up to the point where the swab rod reached.

The second stage took place in the laboratory, where the collected material was seeded into culture media for the growth and isolation of microorganisms. Blood agar, chocolate agar, MacConkey agar, and CLED agar were used as culture media.

In the laboratory, a homogenized culture medium was used to transport the samples using an automatic pipette with a disposable tip, distributed in Petri dishes containing the culture means for the differentiation of microorganisms. Subsequently, using a plastic and sterile bacteriological loop, sowing was performed using successive striations. The plates were then identified with the date, name of the collected surface, and the number that represented the collection, and then incubated in a bacteriological oven at 37 °C. The plates were observed at the following time intervals: 12 h, 24 h, 36 h, 48 h, and 60 h. An electronic colony counter was used to read the plates.

For data analysis, the information was submitted to the appropriate coding and entered into the database through the elaboration of a code dictionary in the Microsoft Excel 2016 worksheet. The growth time of the microorganisms was analyzed according to the microbiological sample collection site and at the second moment using the chi-square test with a significance level of P < 0.05. Univariate analysis, the Shapiro–Wilk test, and, when possible, correspondence analysis, a multivariate tool that analyzes all the variables together, were also performed to optimize the exploratory profile of the data.

## RESULTS

The average time of surgical procedures that used IMV and served as a parameter for data collection in this study was 96.34 min (± 36.52). The minimum and maximum surgical times were 38 and 311 min, respectively.

Table 1 summarizes the types of surgeries performed during the study and their respective frequencies. Gastric bypass surgery for morbid obesity was the most performed, accounting for 10.98% of all procedures, followed by cholecystectomy, local incisional hernia, herniorrhaphy, and breast augmentation, each representing 8.54% of the surgeries. Mastectomy, which accounted for 7.32%, was another notable procedure in terms of frequency.

**Table 1.** Proportions of the type of surgeries performed in the study (n = 82)

Type of surgery	n	%
Gastroplasty for morbid obesity	9	10.98
Cholecystectomy	7	8.54
Local incisional hernia	7	8.54
Herniorrhaphy	7	8.54
Breast augmentation	7	8.54
Breast resection	6	7.32
Tonsillectomy	4	4.88
Glossectomy	4	4.88
Segmentectomy	4	4.88
Thyroidectomy	4	4.88
Video arthroscopic acromioplasty	3	3.66
Benign tumor excision	3	3.66
Exploratory laparotomy	3	3.66
Third ventriculostomy	3	3.66
Laser uretero-reno-lithotripsy	3	3.66
Video cholecystectomy	3	3.66
Spine arthrodesis with instrumentation	2	2.44
Video endoscopy septoplasty	2	2.44
Elbow osteosynthesis	1	1.22

Table 2 shows a significant association between the growth times of the microorganisms in relation to the sample collection site (P < 0.001). The growth of microorganisms occurred within 36 h on most analyzed surfaces, except for the soda lime, which presented a higher growth frequency in 48 h.

**Table 2.** Occurrence of microorganisms in relation to growth time and analysis site (n = 82)

Growing times	Distal portion of the inspiratory branch before anesthesia	Distal portion of the expiratory branch after anesthesia	Breathing circuit canister at the end of anesthesia	Surface area of anesthetic medication (before preparation)	Soda lime
12 h	1 (2.70%)	4 (10.53%)	2 (6.06%)	10 (19.23%)	0 (0.00%)
24 h	14 (37.84%)	14 (36.84%)	10 (30.30%)	12 (23.08%)	0 (0.00%)
36 h	19 (51.35%)	16 (42.11%)	14 (42.42%)	19 (36.54%)	5 (17.24%)
48 h	3 (8.11%)	3 (7.89%)	5 (15.15%)	5 (9.62%)	15 (51.72%)
60 h	0 (0.00%)	1 (2.63%)	2 (6.06%)	6 (11.54%)	9 (31.03%)
P value*	< 0.001				

* P value representing the chi-square test.

Table 3 presents the microorganisms identified at different biological sample collection locations, with a total of 328 samples. The presence of these microorganisms was analyzed in relation to the selected culture medium and sample collection site. Notably, the variation in the occurrence of microorganisms depends on the culture medium and sample collection site. Interestingly, a high level of contamination was observed in certain areas, such as the distal inspiratory part before anesthesia and the anesthetic medication surface before preparation. In all correspondence analyses, the P value was < 0.001 indicating that the observed differences were statistically significant.

**Table 3.** Microorganisms identified in each of the biological sample collection sites (n = 328)

Culture medium	Microorganism	Distal portion of the inspiratory branch before anesthesia	Distal portion of the expiratory branch after anesthesia	Breathing circuit canister at the end of anesthesia	Surface area of anesthetic medication (before preparation)
CLED agar	*E. coli*	35 (42.17%)	41 (41.41%)	28 (42.42%)	41 (22.78%)
	*Enterococcus spp.*	0 (0.00%)	0 (0.00%)	0 (0.00%)	39 (21.67%)
	*Klebsiella spp.*	24 (28.92%)	28 (28.28%)	10 (15.15%)	16 (8.89%)
	*Enterobacter spp.*	0 (0.00%)	0 (0.00%)	3 (4.55%)	39 (21.67%)
	*Staphylococcus aureus* (coagulase negative)	24 (28.92%)	30 (30.30%)	25 (37.88%)	45 (25.00%)
	P value*	< 0.001			
MacConkey agar	*E. coli*	8 (50.00%)	10 (52.63%)	0 (0.00%)	19 (25.00%)
	*Klebsiella spp.*	0 (0.00%)	0 (0.00%)	0 (0.00%)	12 (15.79%)
	*Enterobacter spp.*	8 (50.00%)	9 (47.37%)	0 (0.00%)	18 (23.68%)
	*Proteus* spp*.*	0 (0.00%)	0 (0.00%)	0 (0.00%)	11 (14.47%)
	*Pseudomonas spp.*	0 (0.00%)	0 (0.00%)	0 (0.00%)	16 (21.05%)
	P value*	< 0.001			
Blood agar	*Staphylococcus aureus*	18 (46.15%)	17 (41.46%)	7 (50.00%)	19 (19.39%)
	*Streptococcus pneumoniae*	21 (53.85%)	24 (58.54%)	7 (50.00%)	24 (24.49%)
	*Streptococcus pyogenes*	0 (0.00%)	0 (0.00%)	0 (0.00%)	28 (28.57%)
	*Enterococcus faecalis*	0 (0.00%)	0 (0.00%)	0 (0.00%)	27 (27.55%)
	P value*	< 0.001			
Chocolate agar	*Streptococcus pneumoniae*	18 (100%)	20 (100%)	15 (100%)	39 (75.00%)
	*Neisseria gonorrhoeae*	0 (0.00%)	0 (0.00%)	0 (0.00%)	13 (25.00%)
	P value*	< 0.001			

^a^correspondence analysis.

Table 4 presents the percentages of fungi in the canister, with a total of 47 samples analyzed. This study was conducted to complement bacterial growth research, with a focus on identifying the presence of fungi in biological samples collected from canisters. Of the 82 samples collected, only 9 (10.9%) did not show fungal growth, and *Candida spp*. were the predominant fungus, identified in 24 of the 47 samples, representing an occurrence of 51.06%. This indicates that more than half of the canister samples contained this fungus.

**Table 4.** Percentage of occurrence of fungi in the canister (n = 47)

Microorganism	n	%
*Candida spp.*	24	51.06
*Aspergillus spp.*	7	14.89
*Penicillium spp.*	5	10.64
*Fusarium spp.*	2	4.26
Other fungi	9	19.15

Despite the bacterial growth observed in other parts of the analysis, the soda lime granules did not show microorganism growth. This suggests that although the canister is subject to fungal contamination, the soda lime remains sterilized or free from contamination under the study conditions.

## DISCUSSION

Decreasing the microbial load in the operating room can reduce the risk of surgical wound contamination and general surgical site infections. Sources of environmental contaminants include the skin, hair, and hands of healthcare professionals or the physical environment, such as operating tables, auxiliary tables, and anesthesia machines.^20^

In this study, we observed the growth of the main microorganisms of epidemiological importance at different incubation times and sample collection sites. These results were consistent with the findings of previous studies on the contamination of the inspiratory and expiratory branches^21^ that may be associated with processing that proves ineffective in disinfection.

The processing of corrugated tubes from the inspiratory and expiratory branches of the respiratory circuit of anesthesia machines may become invalid when the norms and protocols recommended by the national and international bodies for processing are not properly followed.^16,21-23^ This reprocessing is performed and conditioned by the human factors responsible for the proper removal of dirt and correct dilution of the products.^16,22-23^

In general, anesthesia machine design makes routine cleaning and disinfection difficult, and complete decontamination is practically impossible in daily practice. Pathogenic microorganisms survive in anesthesia machines after standardized routine cleaning, with the bacterial load reduced but not eliminated even after advanced cleaning practices are initiated.^24-25^

The second possibility of bacterial contamination is the internal handling and storage of reprocessed products. Conducting direct observations in the loco of the reprocessing is necessary to evaluate a series of potential moments that lead to possible failures. Keeping health products unprotected and dry in the air after cleaning and disinfection using a thermodisinfector can influence surface contamination.^25-26^

Our results for the respiratory circuit canister collected at the end of anesthesia, differ from those in the existing literature.^26-27^ In contrast to a previous study that highlighted only the presence of fungi, we observed the growth of both bacteria and fungi at different points in time. A possible explanation^26-27^ is that while the medium in the canister is alkaline, it might not be regularly rinsed. However, certain microorganisms remain viable even under these conditions. Notably, *Candida spp.* was the predominant fungus detected in our study, which has the ability to cause focal invasive infections.^23^

Surface contamination, especially in the area of anesthetic drug preparation before the actual preparation, is alarming. This underscores the importance of cleaning and disinfecting frequently touched surfaces within the anesthesia care area and workspace between procedures using approved hospital disinfectants. The importance of prioritizing surfaces that undergo frequent hand contact, such as drug preparation tables, has been advocated by the global consensus, as noted in various international studies.^28^

Remarkably, our findings demonstrated the consistent presence of *Neisseria gonorrhoeae* on surfaces designated for the preparation of anesthetics and other intravenous drugs, which is rather unusual as the presence of *Neisseria gonorrhoeae* in such an environment is unprecedented in the reviewed studies. Although the exact origin of this microorganism in our samples remains uncertain, its consistent presence is a cause for concern and may indicate lapses in the cleaning process, compromising patient safety. Further investigation is necessary to determine whether this is an anomaly in data collection or indicative of a broader contamination issue.

The study center did not have a protocol for changing the breathing circuit of the anesthesia machine; therefore, certain equipment had HMEF, whereas others did not. These devices are installed between the endotracheal tube and the “Y” connector to reserve part of the heat and steam from expiration, which are available during the inspiration process. The use of this method has several advantages, such as the reduction of gas loss, reduction of water condensation in the respiratory circuit, and cost-efficient.^29-30^ In addition, HMEF is considered efficient in filtering microorganisms; that is, it functions as a physical barrier to protect against microbial contamination, both for patients and mechanical ventilators.

Changing the breathing circuit between patients is recommended to minimize the risk of cross contamination.^27^ However, for the prevention and control of VAP to be efficient, it is necessary to standardize and carry out careful processing of the items that make up this circuit, which was not followed in the study center. The American Association of Nurse Anesthesiology (AANA) recommends that the disinfection process be performed on all components of the anesthesia equipment.^27,31-32^

Ineffective cleaning and disinfection of surfaces is capable of superficially removing the polymeric matrix from the biofilm, which can help release microorganisms of epidemiological importance, such as *Staphylococcus aureus, Staphylococcus pneumoniae,* and *E. coli*.^33^ Formulating a Standard Operating Procedure (SOP) is necessary for the effective execution of this routine. However, evidence to determine the best inputs for use on hospital surfaces is scarce.^34^

In this study, *E. coli and Staphylococcus aureus* were observed in the inspiratory and expiratory branches. However, there was a higher incidence of *E. coli, Enterococcus spp., and Enterobacter spp.* on the surface of the anesthetic medication area. Therefore, it was possible to identify a variety of bacteria and fungi on these surfaces. Despite the numerous potentially pathogenic microorganisms identified, the presence of *Streptococcus pneumoniae,* the most prevalent bacteria implicated in pneumonia warrants special attention.^35-36^

Among the other bacteria identified was *Staphylococcus aureus*, which is the most prevalent clinically relevant infectious agent and a major cause of HAIs. This microorganism can survive for long periods, from 7 days to 7 months, on hospital surfaces.^33^ In addition, most of the fungi identified were of the genus *Candida spp*., which can become an opportunistic agent and cause severe pneumonia in immunocompromised patients, thereby increasing the risk of mortality in such patients.

### Study limitations

Our study had certain limitations. First, this was a single-center study, and therefore the results cannot be generalized. Second, the number of samples collected was influenced by a decrease in surgical procedures owing to the COVID-19 pandemic.

### Contributions to the practice

The microbiological findings of this study indicate that patients on IMV undergoing a surgical procedure may be at a greater risk of developing HAI, reinforcing the importance of standardizing the cleaning, disinfection, and sterilization processes of respiratory circuits.

## CONCLUSION

This study evaluated the contamination of anesthetic medication preparation surfaces, respiratory circuits, and devices used in general anesthesia with assisted mechanical ventilation. These results highlight the importance of ensuring proper cleaning and disinfection of all high-touch surfaces. Contamination by microorganisms can be minimized by creating protocols that define criteria related to the work process, allowing for systematic and frequent analysis of infection prevention and control practices in operating rooms.
